# Burden of herpes zoster in the UK: findings from the zoster quality of life (ZQOL) study

**DOI:** 10.1186/1471-2334-14-402

**Published:** 2014-07-20

**Authors:** Adam Gater, Linda Abetz-Webb, Stuart Carroll, Azharul Mannan, Mick Serpell, Robert Johnson

**Affiliations:** 1 Adelphi Values, Cheshire, UK; 2 Sanofi Pasteur MSD, Berkshire, UK; 3 Gartnavel General Hospital, University Department of Anesthesia, Glasgow, UK; 4 University of Bristol, Bristol, UK

## Abstract

**Background:**

Herpes zoster (HZ) is a painful condition that can have a substantial negative impact on patients’ lives. However, UK-specific data on the debilitating impact of HZ, in terms of patients’ experience of pain and impairments in Health-Related Quality of Life (HRQoL) are limited. The Zoster Quality of Life (ZQOL) study, a large-scale UK cross-sectional study, was conducted to quantify the burden of HZ in UK patients.

**Methods:**

A total of 229 HZ patients aged 50 years or over were recruited from primary and secondary/tertiary care centres throughout the UK. Patients completed a battery of validated questionnaires, including the Zoster Brief Pain Inventory (ZBPI), the Medical Outcomes Study Short-Form 36 (SF-36) and the EuroQol-5 Dimensions (EQ-5D) on initial presentation to the doctor and again 7–14 days later. At follow-up patients also completed the Treatment Satisfaction with Medication (TSQM) questionnaire. Where available, mean questionnaire scores in the HZ population were compared to scores for age-matched norms to investigate the burden associated with HZ.

**Results:**

Pain was prominent among patients, with 57.9% at the initial study visit reporting pain in the preceding 24 hours at levels typically considered to have a significant impact on HRQoL (i.e. ZBPI worst pain ≥ 5). This was reflected in SF-36 and EQ-5D scores that were significantly lower for patients when compared to age-matched norms (p < 0.05) - except for the SF-36 domain of physical functioning. HRQoL was inversely associated with levels of reported pain, with those patients in the greatest amount of pain reporting the greatest HRQoL impact. However, there was no association between pain severity and participant age. The majority of patients (69.4%) received antivirals within 72 hours of rash appearing and 69.9% of patients were also taking analgesics for the management of HZ pain. TSQM scores indicated that patients were least satisfied with the effectiveness of their prescribed treatment.

**Conclusions:**

The acute presentation of HZ is a painful experience that can have a significant impact on the physical and mental wellbeing of sufferers. Findings highlight significant unmet need among patients, particularly in terms of the effectiveness of therapies for the management of HZ.

## Background

Herpes Zoster (HZ), often referred to as ‘shingles’, is a viral condition resulting from reactivation of latent varicella-zoster virus (VZV) which is responsible for childhood ‘chickenpox’ (primary infection). Approximately one in four persons will develop HZ during their lifetime, leading to an estimated 200,000 episodes in the UK annually [1]. Incidence of HZ increases with age, with the majority of cases occurring in patients over 50 years of age [2] and the risk of developing HZ being 50% or more in those aged 80 years and over [3].

HZ manifests in three phases: prodromal, acute and chronic (i.e. long-term complications that may result from HZ). For many patients, each of the phases is characterised by pain. Diagnosis and treatment occur in the acute phase upon the presentation of a unilateral dermatomal rash, which may occur anywhere on the body but most commonly presents on the trunk. The rash may also be present around the nose and eyes (also known as herpes zoster ophthalmicus (HZO)). The dermatological rash and pain associated with HZ typically resolves within one month of presentation [2]. However, despite treatment, some patients may experience long-term complications as a result of HZ. The most common and debilitating complication of HZ is post-herpetic neuralgia (PHN), commonly defined as pain persisting for 90 days or more following HZ rash onset and estimated to occur in approximately 20% of sufferers [4].

Past cross-sectional, epidemiological studies have demonstrated that the pain, and resulting discomfort, associated with HZ and PHN have a substantial and negative impact on patients’ Health-related Quality of Life (HRQoL) and patients’ ability to engage in activities of daily living [5-11]. In addition to the burden encountered by the individual with HZ, the condition is also associated with considerable societal burden. Care provision for HZ and PHN patients, in terms of visits to primary care (general practitioner centres) and outpatient secondary/tertiary care centres (specialist pain clinics and ophthalmologists), inpatient visits (hospitalisations) and prescription costs, for example, is at considerable cost to healthcare systems [12-17]. Furthermore, HZ and PHN are also associated with significant indirect costs, primarily in terms of loss of productivity for patients and caregivers [8,13].

To date, however, only limited real-world evaluation of the burden of HZ and PHN specific to UK patients has been conducted, and what research has been conducted is limited by relatively small sample sizes and a lack of geographic representation [8]. However, UK-specific data are valuable to generate best available evidence for informing national healthcare decisions related to strategies for the prevention and/or management of HZ and PHN. There is a need, therefore, for the generation of more information to accurately ascertain disease burden in UK patients; a view expressed by the UK Joint Committee on Vaccination and Immunisation [18,19].

To address this need, the Zoster Quality of Life (ZQOL) study, the first UK-wide real-world study designed to assess the clinical characterisation, patient-reported burden and wider societal burden associated with HZ and PHN was conducted. HZ-specific information derived from the ZQOL study is outlined herein (ZQOL study findings for PHN patients are reported in a separate manuscript) [20]. Specifically, the following information and implications for such findings are considered: (1) disease presentation in UK HZ patients (2) burden of HZ for UK patients aged 50 years and over in terms of subjective experience of pain and HRQoL impact (3) economic burden associated with HZ in terms of work loss productivity and healthcare resource (e.g. prescribed medical interventions, time spent with healthcare professionals etc.) (4) HZ patient satisfaction with prescribed medical interventions.

## Methods

### Study overview

**Figure 1:**
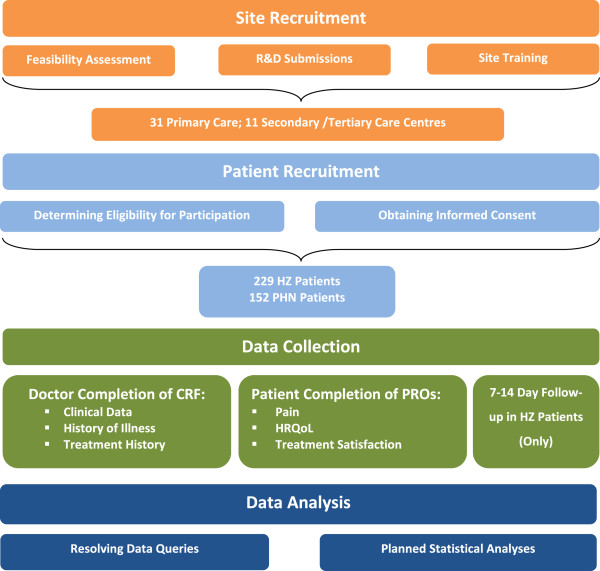
Overview of study methodology.

The ZQOL study adopted a cross-sectional observational design involving data collection from HZ/PHN patients and their treating doctors (Figure 1). No medical product or device was administered to study patients, nor did participation in the study necessitate any change from standard of care.

### Recruitment

A total of 230 HZ patients were targeted for recruitment to the ZQOL to ensure sufficient power for pre-specified primary statistical analyses (i.e. comparison of patient-reported outcome data collected from study collected from study participants to published age-matched normative values for the respective instruments). This sample size was verified via 100,000 Monte Carlo simulations, using the Hochberg (1988) Step-up Procedure to control for family-wise error rate in multiple comparisons [21]. As a reflection of the typical management of HZ, ZQOL study patients were recruited via incident case referrals from primary and secondary/tertiary care centres, throughout England, Scotland, Wales and Northern Ireland [22].

Consecutive patients presenting with HZ at the site as part of routine clinical practice were considered for participation in the study. Patient eligibility for participation in the study was ascertained by doctor completion of a study-specific case report form. To be eligible for participation in the ZQOL study patients were required to present to the doctor with a visible HZ rash (i.e. unilateral crop of blisters and scabs in a dermatomal distribution). Furthermore, in accordance with data suggesting that the incidence and severity of HZ increases significantly in adults over the age of 50 [2], only patients within this age range were eligible for participation in this study.

To promote the ecological validity of the study (i.e. the extent to which study setting, methods and materials approximate the real-world that is being examined), extensive exclusion criteria were not employed. However, to protect the integrity of study findings, HZ patients with health impairments that may make it difficult for them to complete the required battery of PRO instruments (e.g. those with deficits in cognitive functioning or visual impairments) were excluded from participation. Similarly, patients whose medical background may make it difficult to interpret study results (e.g. patients who have taken part in a clinical trial related to HZ, pain and/or immunomodulating therapy in the past 6 months or patients who were previously experiencing neuropathic pain in the dermatomal region of their HZ rash prior to the onset of HZ due to another aetiology) were also excluded. Of note, PRO instruments were only provided in English and non-English speakers were excluded from participation in the study.

### Study procedures

Prior to recruitment of study patients, all site staff received formal training in study procedures, including procedures for administering Patient-Reported Outcome (PRO) questionnaires to patients to ensure standardisation and minimisation of missing data (e.g. ensuring that patients do not receive additional help from anyone when completing the questionnaires and that questionnaires are thoroughly checked for completeness). In addition, as a pre-requisite of site participation, all site staff were required to demonstrate evidence of completion of good clinical practice training.

At the initial site visit, once informed consent had been obtained and participant eligibility for participation confirmed, patients were asked by study staff to complete a combination of validated and specially designed PRO questionnaires. All questionnaires were bound in a study booklet to ensure standardisation of presentation and order of completion among all patients. Participants were informed prior to obtaining consent that completion of the PRO questionnaires should take no more than 30 minutes.

There is no consensus in the research literature as to the stage of the acute HZ episode at which pain and HRQoL impact are at their highest [6,23]. Therefore, to ensure that the patient-reported impact of acute HZ was not underestimated by data collection on initial presentation, all patients were provided with additional copies of the validated PRO questionnaires which they were asked to complete at home and return 7–14 days after their original visit.

### Study questionnaires

Data were collected using the following PRO questionnaires:

•**Patient socio-demographic questionnaire:** Used to collect patient socio-demographic data (age, gender, ethnicity, living situation, education level and employment status) and data concerning patients’ experiences of HZ (e.g. symptoms during the acute phase of disease presentation). In addition this questionnaire also included questions concerning the impact of shingles on patients’ daily lives (e.g. impact on work productivity, number of days unable to work/take part in daily activities such as housework and hobbies). Details of medical care (e.g. number of doctor consultations) and treatment (e.g. use of non-prescription drugs) for shingles were also collected. Completed at initial visit only.

•**Zoster Brief Pain Inventory (ZBPI)**[24]**:** The ZBPI is designed specifically to evaluate pain intensity due to HZ (which may include allodynia and pruritus) and the extent that this may interfere with respondent’s activities of daily living. The instrument comprises 15 items taken from the Brief Pain Inventory-Short Form, a well-validated PRO measure applied in a broad spectrum of medical conditions [25] and includes four items assessing ‘Pain severity’ and seven items assessing ‘Pain interference’. All items comprising these subscales are assessed via 11-point numerical rating scales ranging from 0 (no pain/does not interfere) to 10 (pain as bad as you can imagine/completely interferes). Prior research has shown the ZBPI to be a reliable and valid assessment of pain severity and impact for use in HZ patients [24] and the instrument has been utilised in prior cross-sectional investigations [5,6,8]. Completed at initial visit and follow-up.

•**MOS 36-item Short-Form Health Survey (SF-36) Acute Form**[26]**:** The SF-36 is a standardised generic questionnaire that consists of 36 items evaluating HRQoL. The psychometric validity of the SF-36 is well-established. In particular, the SF-36 has demonstrated validity for use in HZ and PHN populations [10,11]. Items explore the following eight dimensions: physical functioning, role functioning due to physical problems, role functioning due to emotional problems, bodily pain, general health perceptions, vitality, social function and mental health. Two summary scores, the Physical Component Summary (PCS) and Mental Component Summary (MCS), can also be calculated. The acute form differs from the standard form of the SF-36 in that it employs a recall period of ‘during the past week’ (rather than ‘during past 4 weeks’) which is more appropriate for short-term conditions such as HZ. **S**cores for the eight scale dimensions and two summary scores were calculated using norm-based scoring (NBS) algorithms which employ a linear T-score transformation with mean = 50 and standard deviation = 10 – making it possible to meaningfully compare scores across domains/summary scores [27]. Completed at initial visit and follow-up.

•**The EuroQoL 5 Dimensions questionnaire (EQ-5D)**[28]**:** The EQ-5D is a standardised generic questionnaire comprising 5 items that are used to provide a simple descriptive profile and a single index value for health status (ranging from 0-1): EQ-5D Health State Index (HSI). In addition, the EQ-5D also includes a patient-completed visual analogue scale, which records the respondent’s self-rated health (SRH) on a vertical scale where the endpoints are labelled from 0 (‘worst imaginable health state’) to 100 (‘best imaginable health state’). Completed at initial visit and follow-up.

•**Treatment Satisfaction Questionnaire for Medication (TSQM) Version II**[29]**:** The TSQM Version II is an 11-item questionnaire designed to assess patients’ satisfaction with various aspects of their medication, including side effects (3 items + 1 yes/no stem), effectiveness (2 items), convenience (3 items) and overall treatment satisfaction (2 items). Items are assessed using 5-point and 7-point Likert scales ranging from ‘Extremely dissatisfied’ to ‘Extremely satisfied’, but scores on all domains are transformed to a 0-100 scale whereby higher scores indicate greater levels of patient satisfaction. Completed at follow-up only.

To complement and facilitate interpretation of PRO data, data concerning patient’s clinical and treatment history were collected as part of the doctor-completed case report form.

### Ethics

The ZQOL study was designed and conducted in accordance with the principles set forth in the Declaration of Helsinki [30]. In accordance with current research governance frameworks in the UK, ethical approval of the study was obtained from the National Research Ethics Service before data collection began. Furthermore, as the study involved use of National Health Service (NHS) staff, premises, resources and data, local R&D management approval was obtained from the respective NHS local authorities before research began at each site [31]. Finally, informed consent was obtained from patients prior to the collection of data from themselves or their doctor and all data were handled in accordance with the 1998 Data Protection Act.

### Statistical analyses

All planned statistical analyses were specified a priori in a formally developed Statistical Analysis Plan (SAP). All study data were subject to extensive quality control checks prior to the conduct of analyses. Where doctor-reported information was not clear, follow-ups with doctors were made to resolve queries. In accordance with developer instructions, if missing data for a SF-36 domain were < 50% then data were imputed using the mean of participants’ responses to the completed items [26]. Missing data for the ZBPI, SF-36 (where >50%), EQ-5D and TSQM were not imputed and where item scores were missing, relevant domain scores were not calculated.

To quantify the burden associated with HZ, mean SF-36 and EQ-5D scores from ZQOL study samples were compared to published age-matched normative values for the respective questionnaires [32,33]. The statistical significance of differences was investigated via the conduct of unifactorial tests. Differences of equal to or greater than 0.5 standard deviation units of a baseline or comparator score were characterized as clinically meaningful [34-36].

In addition, a number of sub-analyses were conducted upon patient scores on the composite domains of the ZBPI (pain severity and pain interference), SF-36 (PCS and MCS) and EQ-5D (HSI and SRH) to test a number of pre-specified hypotheses, including:

Age: Prior research has indicated HZ disease severity increases with age [37]. The association between patient age and reported pain severity, pain interference and HRQoL impact was investigated via calculation of Spearman Rho correlation coefficients.

Gender: In light of previous evidence suggesting differential reports of pain severity, pain interference and HRQoL impact by gender across a range of medical conditions (including HZ) [38,39], mean composite scores among male and female patients were compared using unifactorial tests.

Antiviral use: There is evidence to suggest that use of antivirals within 72 hours of rash onset can reduce the severity and duration of the HZ episode [40-42]. To account for any potential differential impact, therefore, follow-up data for patients prescribed an antiviral within 72 hours of rash onset were compared to those who received an antiviral more than 72 hours after rash onset.

Pain severity (ZBPI ‘Worst pain’): It was expected that pain interference and HRQoL impact would be greatest among patients reporting the greatest levels of pain. Pain interference and HRQoL were therefore to be compared amongst groups of patients stratified according to empirically confirmed categorisations of scores on the ‘Worst pain’ item of the ZBPI: none (0); mild (1–4); moderate (5–6); severe (7–10) [43]. ZBPI ratings of ‘Worst pain’ were considered as past research has indicated that ratings of ‘worst pain’ are the more reliable than ratings of ‘average’ or ‘current’ pain [24]**.**

In order to explore potential predictors of pain and HRQOL (as assessed by composite scores for the aforementioned PRO questionnaires), a series of ordinary least squares (OLS) regression analyses were conducted. Independent predictor variables included: socio-demographic variables (age, gender, ethnicity, BMI etc.); time elapsed since first appearance of rash; diagnosis of HZO; antiviral prescription within 72 hrs of rash presentation; level of analgesics used (level 1 vs. level 2 vs. level 3); hospitalisations (binary – Yes/No); Co-morbidities; and ZBPI ‘Worst pain’ item scores.

## Results

### Socio-demographic characteristics

A total of 229 HZ patients (214 HZ, 15 HZO) were recruited to the ZQOL study between April 2010 and May 2011 (Table 1). Both age [5,12] and gender distributions [5,6,10-12] of the study sample are consistent with prior epidemiological investigations of the incidence of HZ in the UK and previous studies in the US, Canada and Europe. Of further note, reports from study sites suggest that very few patients approached were deemed ineligible for participation in the study.

**Table 1:** HZ ZQOL study patients: socio-demographic data

**Characteristic**	**HZ patients (n = 229)**
**Age years – mean (Range)**	67.6 (50 – 94)
**Gender n (%)**	
Male	105 (45.9%)
Female	122 (53.3%)
Not stated	2 (0.9%)
**Body Mass Index (BMI) – mean (Range)**	27.7 (19 – 49)
**Ethnicity n (%)**	
White/Caucasian	225 (98.3%)
Mixed race	1 (0.4%)
Not stated	3 (1.3%)
**Comorbidities n (%)**	
Any comorbid disorder	186 (81.2%)
Cardiovascular	117 (51.7%)
Immune	7 (3.1%)
Metabolic/endocrine	51 (22.3%)
Neoplasms	28 (12.2%)
Psychological/psychiatric	35 (15.3%)
Respiratory	27 (11.8%)
Rheumatoid/neurological	66 (28.8%)
**Highest level of education n (%)**	
Secondary school or less	88 (38.4%)
O level or equivalent	48 (21.0%)
A level or equivalent	16 (7.0%)
Vocational	44 (19.2%)
Undergraduate degree	15 (6.6%)
Post graduate degree	13 (5.7%)
Not stated	5 (2.2%)
**Work status n (%)**	
Full or part time employment	66 (28.8%)
Retired	146 (63.8%)
Unable to work for medical reasons	9 (3.9%)
Unemployed	3 (1.3%)
Mature student	1 (0.4%)
Other	2 (0.9%)
Not stated	2 (0.9%)
**Living situation n (%)**	
Living alone	45 (19.7%)
Living with partner	148 (64.6%)
Living with partner and children	20 (8.7%)
Living with children	7 (3.1%)
Living in communal residence (e.g. care home)	2 (0.9%)
Other	5 (2.2%)
Not stated	2 (0.9%)

### Characterisation of HZ episode

For 79.9% (n = 183) of HZ patients, this was their first episode of the disease. Among patients who had experienced HZ before, the majority had experienced just one prior episode. That 20.1% (n = 46) of patients were experiencing a recurrent episode of HZ (albeit based on patients’ own reports and not confirmed clinical diagnoses) suggests that HZ is not a once in a lifetime event and supports recent evidence indicating that recurrent episodes of HZ are more common than previously reported [44].

**Figure 2:**
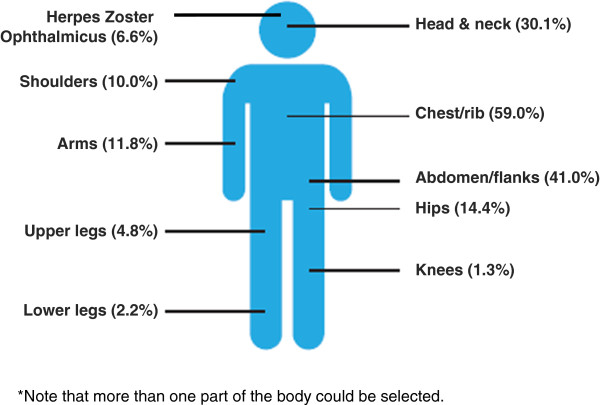
Area of body affected by HZ among ZQOL study patients (n = 229).

Almost all patients (97.4%; 223) were described by their physicians as presenting with classic HZ (i.e. a unilateral crop of blisters in a dermatomal distribution). The majority of HZ rashes were described by doctors as being erythematous (72.1%; n = 165) or simple vesicles (62.9%; n = 144) in appearance, of a small size (48.5%; n = 111) and a medium density (54.1%; n = 124). Among ZQOL study patients, the HZ rash most commonly affected more than one site of the body (60.3%; n = 138) and most frequently presented on the chest/rib cage (59.0%; n = 135), abdomen/flanks (41.0%; n = 94), and head and neck (30.1%; n = 69) (Figure 2). Of note, despite concerted efforts to recruit such patients to the study via ophthalmologists, the proportion of patients with HZO patients recruited to the ZQOL study (6.6%; n = 15) was less than projected population estimates (10-20%) [22].

The vast majority (90.4%; n = 207) of HZ patients reported experiencing pain due to their HZ rash. Reports from patients indicated that this pain was persistent in nature, being present ‘Most of the time’ (44.1%; n = 101) or ‘All the time’ (15.3%; n = 35). The experience of pain following HZ rash onset was qualitatively similar to that experienced prior to rash onset with ‘tender’ (52.0%; n = 119), ‘sensitive’ (40.6%; n = 93), ‘itching’ (38.4%; n = 88), ‘burning’ (35.8%; n = 82) and ‘aching’ (33.6%; n = 77) emerging as the terms most frequently selected by patients to describe their pain.

In addition to pain, findings from the ZQOL study reveal that HZ patients also experience a range of other symptoms during the acute phase of HZ. In particular, symptoms of fatigue (70.7%; n = 163), stomach upsets (30.6%; n = 70), change in bowel movements (27.1%; n = 63) and muscle weakness (27.1%; n = 63) were commonly experienced by study patients. This is in contrast to prior research which suggests that such symptoms occur in <20% of patients [23].

### Quantification of pain experienced by HZ patients: ZBPI

**Figure 3:**
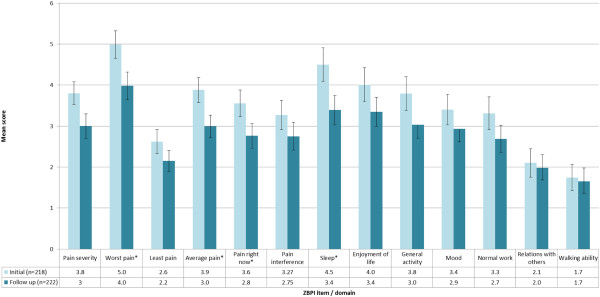
**ZBPI domain and component scores among HZ ZQOL study patients at initial visit and follow-up.** No *s p≥0.05, *p<0.05.

ZBPI scores from ZQOL study patients indicated that pain severity and pain interference, as assessed by ZBPI composite scores and individual ZBPI items, were highest on initial presentation to the recruiting doctor with a visible HZ rash than compared to 7–14 days later at follow-up (Figure 3). During this initial visit more than half of all HZ patients (57.6%; n = 132) gave ratings of ‘Worst pain’ experienced in the past 24 hours that are indicative of significant HRQoL burden (i.e. worst pain score ≥ 5).

Comparison of patient ratings of pain as defined according to empirically established cut-offs for the ZBPI worst pain item (1–4 = mild pain; 5–6 = moderate pain; 7–10 = severe pain) [45] and doctor ratings of pain (mild, moderate and severe) indicated agreement in only 40.6% of cases. Where disagreement occurred, doctors were more likely to underestimate (35.6%) than overestimate (23.7%) patients’ experience of pain. These findings are consistent with previous research that has indicated a disparity between patient and doctor and assessments of pain [46].

In contrast to prior research suggesting a link between HZ pain severity/interference and age [37], calculation of Spearman rho correlation coefficients revealed no significant association between patients’ age and composite pain severity and pain interference scores (Table 2). Investigation of ZBPI pain scores revealed that females scored significantly higher than males in terms of pain severity. Differences for pain interference, however, were non-significant (Table 2). Further subgroup analyses revealed pain severity and interference to be highest among patients prescribed antivirals within 72 hours of rash, compared to those not prescribed antivirals within this timeframe (although only differences for pain interference were statistically significant). This may reflect that patients with high levels of pain are more likely to consult with their doctor early and be prescribed antivirals within 72 hours or rash onset. As expected, a significant linear relationship was also observed in mean pain interference scores for patients classified according to empirically confirmed cut-offs for pain severity (Table 2).

**Table 2:** HZ pain severity/interference (ZBPI) and HRQOL impact (SF-36 & EQ-5D) at initial visit: ZQOL study subgroup analyses

**Outcome variable**	**Age**		**Gender**			**Antiviral**			**Pain Severity**				
	**Spearman’s rho**	**P**	**Male (n = 105)**	**Female (n = 122)**	**P**	**<72 hours (n = 159)**	**≥ 72 hours (n = 43)**	**p**	**No pain (n = 23)**	**Mild (n = 66)**	**Moderate (n = 48)**	**Severe (n = 79)**	**p**
**Zoster Brief Pain Inventory (ZBPI)**													
Pain Severity	−0.0720	NS	3.4	4.0	*	3.9	3.4	NS	N/A	N/A	N/A	N/A	N/A
Pain Interference	−0.0173	NS	3.1	3.4	NS	3.5	2.6	*	0.7	1.7	3.5	5.3	*** ^†^
**Short-Form Health Survey 36 (SF-36)**													
SF-36 Physical Component Summary (PCS)	−0.0903	NS	40.9	42.1	NS	43.2	46.2	NS	48.3	44.8	41.0	36.07	***
SF-36 Mental Component Summary (MCS)	0.0840	NS	45.9	39.9	*** ^†^	41.5	46.9	NS	46.4	45.5	38.5	41.1	**
**EuroQoL 5-Dimensions (EQ-5D)**													
EQ-5D Self-Rated Health (SRH)	−0.0295	NS	66.7	59.6	**	63.8	73.1	NS	70.0	70.1	59.7	54.6	***
EQ-5D Health State Index (HSI)	−0.0635	NS	0.68	0.61	NS	0.63	0.70	NS	0.81	0.70	0.66	0.52	***

NS p ≥ 0.05, *p < 0.05; **p < 0.01; ***p <0.001. † = clinically meaningful difference.

Prediction of ZBPI composite scores via ordinary least squares regression analyses revealed that patient use of level 2/3 analgesics (*r*^2^ = 0.065), patient BMI (*r*^2^ = 0.043), the length of time since the patient’s HZ rash first appeared (*r*^2^ = 0.030) and presence of a respiratory disorder (*r*^2^ = 0.023) were able to explain 14.2% of the variance in ZBPI pain severity composite scores. Similar regression analyses revealed that patient ratings of ‘worst pain’ (*r*^2^ = 0.268), use of level 2/3 analgesics (*r*^2^ = 0.024) and presence of a respiratory disorder (*r*^2^ = 0.016) accounted for 36.0% of the variation in pain interference scores.

### HZ impact on HRQoL: SF-36

**Figure 4:**
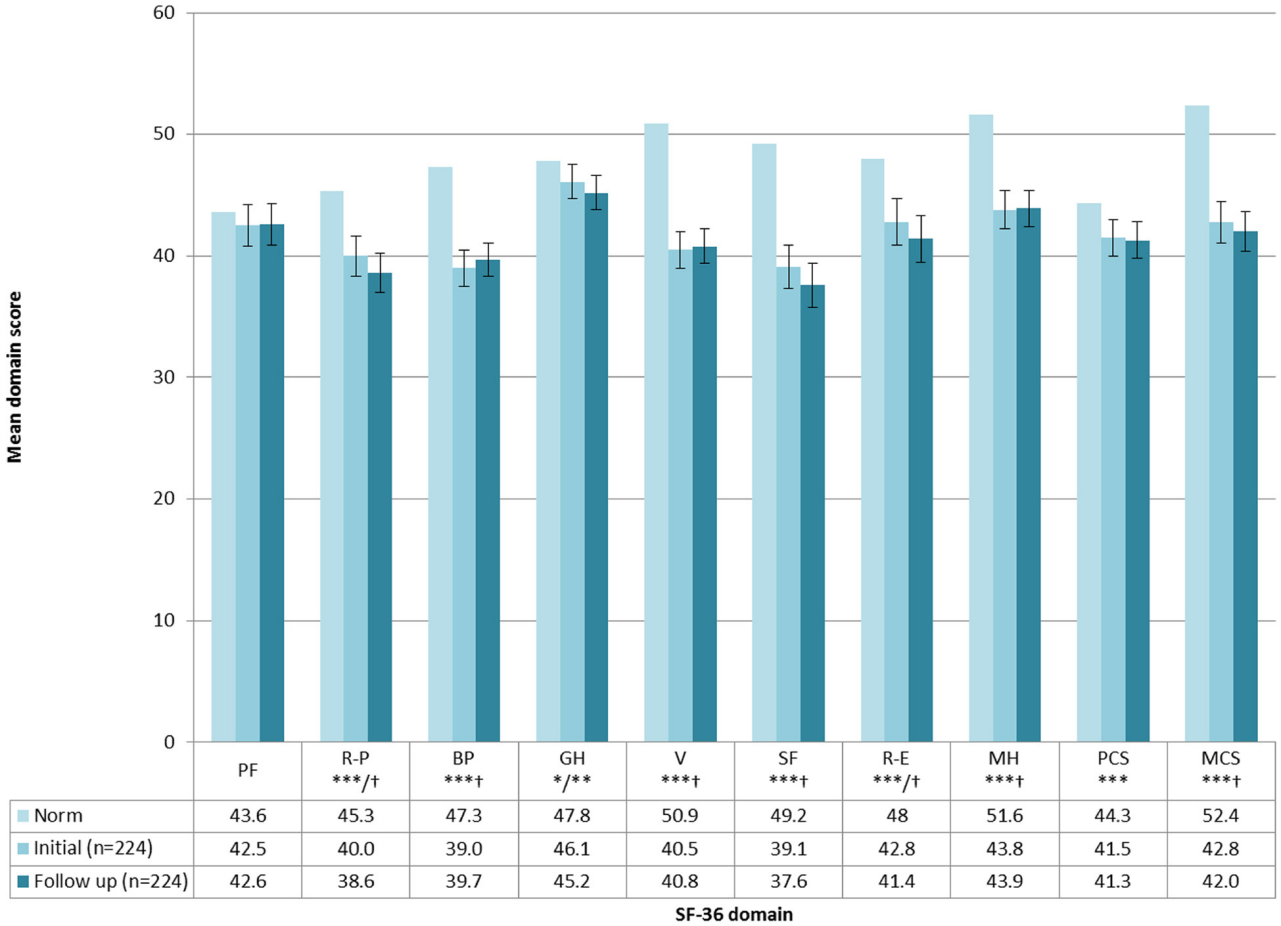
**SF-36 domain and component scores among HZ ZQOL study patients at initial visit and follow-up.** No *s p≥0.05, *p<0.05; **p<0.01; ***p <0.001. † = clinically meaningful difference. PF=Physical functioning, R-P=Role-physical, BP=Bodily pain, GH=General health, V=Vitality, SF=Social functioning, R-E=Role-emotional, MH=Mental health, PCS=Physical component summary, MCS=Mental component summary.

HZ patients demonstrated significant deficits on all SF-36 domain and summary scores at both initial and follow-up visits (except for Physical functioning) compared to values derived from an age-matched normative population (Figure 4). Deficits, compared to norm values, were generally greatest for SF-36 domains assessing mental functioning, with clinically meaningful deficits observed for domains of ‘Vitality’, ‘Social-functioning’ , ‘Role-emotional’ (follow-up only),  ‘Mental health’ and the MCS.

Calculation of spearman rho correlation coefficients revealed no significant association between patients’ age and PCS and MCS scores. Significant differences were also not observed between subgroups of HZ patients defined according to antiviral use. Differences in PCS and MCS scores were observed between male and female HZ patients, with females scoring lower on both domains. However, differences were only statistically significant for MCS scores. A significant linear relationship between PCS and MCS scores was observed for patients classified according to empirically confirmed cut-offs for pain severity (Table 2).

Ordinary least squares regression analyses revealed the presence of rheumatoid/neurological disorders (*r*^2^ = 0.061), patient ratings of ‘worst pain’ (*r*^2^ = 0.048), use of level 2/3 analgesics (*r*^2^ = 0.043), patient BMI scores (*r*^2^ = 0.035) and the presence of a respiratory disorder (*r*^2^ = 0.021) collectively explained 27.2% of the variance in PCS scores. Similar regression analyses revealed that patient ratings of ‘worst pain’ (*r*^2^ = 0.039), patient’s gender (*r*^2^ = 0.033), prescription of antiviral medication within 72 hours of the onset of the HZ rash (*r*^2^ = 0.029) and presence of a psychological/psychiatric disorder (*r*^2^ = 0.023) accounted for 12.1% of the variance in MCS scores.

### HZ impact on HRQoL: EQ-5D

**Figure 5:**
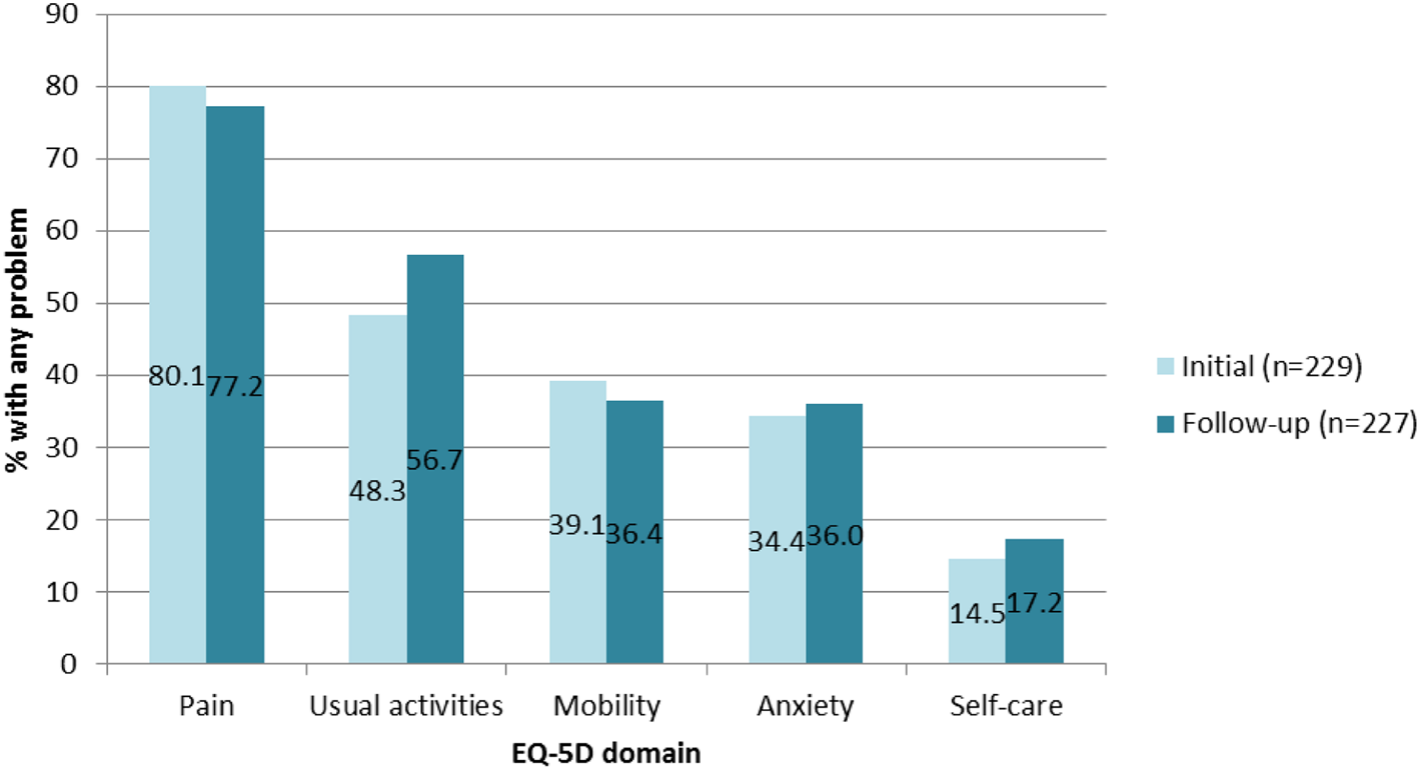
Per cent of HZ patients reporting any problem on EQ-5D domains at initial visit and follow-up.

Consideration of individual EQ-5D scores indicated that ‘Pain’ was the most prevalent problem reported by patients (>80%) at both the initial visit and at follow-up (Figure 5). Mean scores on the Self-rated health (SRH) and Health state index (HSI) were marginally lower at the initial visit when compared to the follow-up visit (differences non-significant). At both visits, however, SRH and HSI scores were significantly lower than the age-matched norms (Table 3).

**Table 3:** Comparison of EQ-5D SRH and HSI scores at initial visit and follow-up to age-matched population norms

**Outcome variable (n)**	**Norm**	**Initial (p)**	**Follow-up (p)**
EQ-5D SRH: Self-Rated Health (229)	77.3	62.4***^†^	66.6***^†^
EQ-5D HSI: Health State Index (227)	0.78	0.65***^†^	0.66***

***p <0.001. † = clinically meaningful difference.

Calculation of spearman rho correlation coefficients revealed no significant association between patients’ age and SRH and HSI scores. Significant differences were also not observed between subgroups of HZ patients defined according to antiviral use. Differences in SRH and HSI scores were observed between male and female HZ patients, with females scoring lower on both domains. Differences, however, were only statistically significant for SRH scores. A significant linear relationship between SRH and HSI scores was observed for patients classified according to empirically confirmed cut-offs for pain severity (Table 2).

Ordinary least squares regression analyses indicated that the presence of rheumatoid/neurological (*r*^2^ = 0.049), psychological/psychiatric (*r*^2^ = 0.034), or ‘uncategorised’ disorders (*r*^2^ = 0.016), the ‘Worst pain’ experienced (*r*^2^ = 0.085) and patient use of level 2/3 analgesics (*r*^2^ = 0.021) explained 22.0% of the variance in SRH scores. The presence of a psychological/psychiatric (*r*^2^ = 0.025), rheumatoid/neurological (*r*^2^ = 0.043), the ‘Worst pain’ experienced (*r*^2^ = 0.041) and patient use of level 2/3 analgesics (*r*^2^ = 0.073) explained 20.7% of the variance in HSI scores.

### Productivity losses in HZ patients

Of the 63/229 HZ patients that were working, 39 (61.9%) reported that their work had been affected by their HZ with 32 of these patients having to take time off work as a result of their HZ. Among those patients who reported that their work had been affected by their HZ, the average number of days off work was 5.4 days (range = 0–28). Reports from the entire HZ patient sample indicate that the mean number of days that they were unable to take part in their usual activities was 3.9 days (range = 0–21).

### Impact of HZ on medical resource use

Clinician reports indicated that HZ patients were taking an average of four medications to treat their HZ (3.89, SD = 1.01). Antiviral therapies (predominately acyclovir) were the most common treatment prescribed by doctors for the management of HZ (88.2%; n = 202) with the majority of patients (69.4%; n = 159) prescribed antivirals within 72 hours of the onset of their rash, the timeframe in which they have demonstrated efficacy [40-42]. Prescription analgesics (including Level 1, Level 2, Level 3, local anaesthetic/analgesics and topical analgesics) and non-prescribed analgesics (including paracetemol and ibuprofen) were also widely used by patients (71.2% and 41.9% respectively). For the majority of patients this was the first time that they had seen a healthcare professional in relation to their HZ (87.3%; n = 200). Follow-up visits were only anticipated for 25.8% (n = 59) of patients. Planned referrals to other healthcare professionals (3.5%; n = 8) and additional investigations (4.4%; n = 10) were low and only one patient reported being hospitalised due to their HZ (due to central nervous system complications).

### Treatment satisfaction among HZ patients (TSQM)

**Figure 6:**
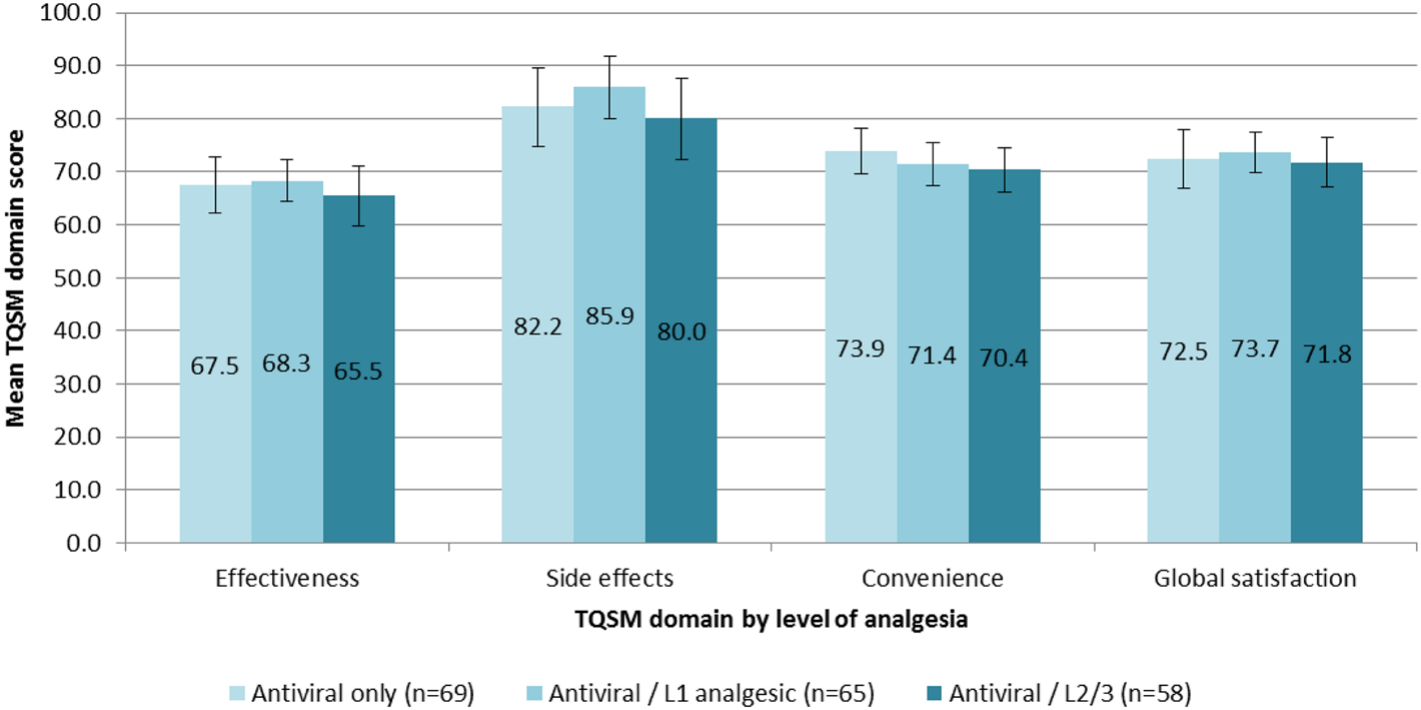
**TSQM domain scores among HZ study patients, categorized according to prescribed treatment.** Note that due to small sample sizes, results for patients receiving no antiviral in combination with level 1 analgesics (n=6) or level 2 analgesics (n=5) have not been presented.

HZ patients were classified according to antiviral and analgesic medication use. Across all groups, TSQM scores indicated that patients were least satisfied with the perceived effectiveness of treatment and most satisfied with the side effects experienced. No significant or clinically meaningful differences were observed between patients receiving different treatment for the management of their HZ (Figure 6).

## Discussion

The value of real-world data summarising patients’ experiences of living with a health condition in informing decisions regarding the management of that condition and access to new medicines in national markets are becoming increasingly recognised. As stipulated in the UK Government’s Health and Social Care Act, the proposed commissioning outcomes framework (which is a fundamental foundation of the proposed NHS reforms in England) will place a strong emphasis on promoting decision-making that is driven by the patient experience; as likely measured by PRO questionnaires [47]. Conducted against the backdrop of a challenging environment for UK observational research [48], the ZQOL study is the first UK-wide study designed to investigate the burden and unmet needs among HZ patients in the UK.

### Understanding the experience of pain and HRQOL burden in UK HZ patients

Results from the ZQOL study indicated that more than half of study participants with HZ (57.9%) reported pain at levels typically considered indicative of significant HRQOL burden [24]. There is limited consensus on the stage at which pain during an acute HZ episode is at its highest [6,23]. However, findings from the ZQOL study indicated that pain was greater on initial presentation to the doctor than compared to follow-up 7–14 days later. That the pain associated with HZ may result in significant HRQoL impairments among HZ patients is well-documented [5-11]. Only rarely in literature published to date, however, has the degree of burden experienced by HZ patients been interpreted with reference to HRQoL readings among normative populations [11]. Comparison of scores on standard generic measures of HRQoL (SF-36 and EQ-5D) to those elicited from age matched normative populations revealed significant deficits across all facets of HRQOL among HZ patients. Deficits among participants were most notable among the mental/affective components of HRQoL and participants ability to perform usual activities where statistically significant and clinically meaningful deficits were observed.

Consistent findings across the PRO measures employed in the ZQOL study were observed. For example, that a high proportion of patients experienced problems conducting usual activities (as measured by the EQ-5D) is consistent with significant and clinically meaningful deficits observed for SF-36 domains of Role-Physical and Social Functioning and ZBPI ratings of interference on general activities. Similarly, reports of high levels of interference in sleep (as measured by the ZBPI) are consistent with statistically and clinically meaningful deficits observed for the SF-36 Vitality domain. Furthermore, that problems with Self-Care were least frequently reported by participants completing the EQ-5D is also consistent with SF-36 scores for Physical Functioning where statistically significant deficits compared to norms were not observed. Statistically and clinically meaningful deficits observed for the SF-36 domains of Role-Emotional and Mental Health, however, indicate that the mental impact of HZ is likely to extend beyond anxiety and mood (as measured by the EQ-5D and ZBPI, respectively).

Investigation of the burden of HZ in this manner is important for understanding the value of therapies in a given disease area and for facilitating decisions regarding reimbursement. This is particularly true for countries (including the UK), where health authorities may have to determine allocation of finite resources across different diseases. The introduction of Value-Based Pricing (VBP) of medicines into the NHS in 2014, for example, will mean increased emphasis being placed on the generation of robust real-world evidence to support pricing and reimbursement decisions [49].

Limitations on available resources often mean that there is pressure on health providers to target resources to those patients with the greatest health needs. Consistent with hypotheses of a linear relationship between pain severity, pain interference and HRQoL impact, patient-reported levels of pain severity were found to the most consistent and most significant predictor of HRQoL burden. Specifically, the greatest levels of burden were observed in those patients experiencing the most severe levels of pain [6,7,9,10]. However, whilst the prevalence of HZ may be associated with age, findings from the ZQOL study indicate that pain severity and subsequent HRQOL are not predicted by age. Consistent with prior research, however, findings did suggest that pain severity and associated deficits in HRQoL were greatest among female patients. Differences between males and females, however, were not significant in all cases [38,39].

### Economic burden of HZ

Prior research findings from retrospective reviews of medical records and databases indicate that care of HZ patients is associated with significant direct and indirect costs [12,14-16,50]. Direct costs associated with treatment of HZ (including medication costs, doctor visits and hospital admissions) equate to approximately £198 per episode [51]. This seemingly low cost is likely a reflection of the acute presentation of the disease. This is supported by data from the ZQOL study which suggests that, during this time period, resource use among HZ patients is relatively low. For example, beyond the initial consultation, the majority of HZ patients in the ZQOL study (73.4%) were unlikely to be seen again by their doctor in relation to their HZ. Furthermore, rates of referrals to healthcare professionals, additional investigations and hospitalisations as a result of HZ (during initial presentation) were all very low (Note that the long-term implications of HZ are documented in the PHN ZQOL sub-study) [20]. Findings from the ZQOL study, however, indicate that significant losses in productivity may be associated with HZ. This is particularly true among those sufferers not yet at retirement age. For example, two thirds (39/63) of ZQOL patient with HZ and currently in full or part-time employment noted that their work had been affected by their condition, predominately in terms of time taken off work. Previously published research has indicated that, when productivity losses are taken into account, the cost per episode of HZ can be as much as £526 [8].

### Treatment evaluation

Existing guidelines for healthcare professionals recommend that all HZ patients ≥50 years be prescribed antivirals for the management of their condition [52]. Consistent with recommendations, antivirals were the most common treatment prescribed to ZQOL study participants, followed by World Health Organization (WHO) level 1/2/3 and topical analgesics. Whilst the efficacy of antiviral and analgesic medications has been confirmed in the context of clinical trials, there have been only limited attempts to conduct real-world evaluations of these products from the patients’ perspective. Patients’ satisfaction with treatment is an important indicator of treatment outcomes, not least because it is a significant predictor of patients’ adherence to treatment regimens. To date, however, there has been no attempt to quantify patient satisfaction with HZ medications using standardised assessments of treatment satisfaction. Consistent with assertions that antiviral and analgesic medications are generally well-tolerated, analysis of TSQM scores suggested that patients were most satisfied with the side-effects of their treatment. Regardless of the treatment received, lowest levels of satisfaction were reported in relation to the efficacy of the prescribed treatment. This is perhaps not surprising given that the majority of patients were experiencing pain -- to a clinically significant degree in a substantial proportion of patients -- despite receiving treatment for their HZ.

### Implications of ZQOL study findings for management of HZ in the UK

Findings from the ZQOL study have implications in terms of provision of frontline services for HZ and patients and wider implications in terms of the national availability of means to prevent and manage HZ.

As life expectancy throughout the developed world continues to rise, effective management of HZ becomes increasingly important in order to minimise the individual patient and societal burden associated with these conditions. Findings from the ZQOL study support the notion that, in order to address the unmet needs prevalent among HZ patients and to reduce the economic burden associated with HZ, there is a need to move away from therapies designed to manage the symptoms of HZ. Rather, more targeted therapies designed to reduce the burden of HZ by preventing VZV reactivation and attenuating pain severity during the HZ rash and persistence of pain following the resolution of the HZ rash are needed. This is in line with worldwide health authority initiatives, which have stressed the prioritisation of preventative measures, so as to reduce the burden on healthcare conditions on individuals, healthcare systems and society as a whole [53]. Furthermore, this is also in line with the Quality, Innovation, Productivity and Prevention (QIPP) initiative recently introduced by the UK Government [54].

Recent years, have seen documented evidence of the efficacy of a VZV vaccine in the prevention of HZ and PHN episodes and attenuation of the severity of HZ and PHN episodes and associated impact [55]. The VZV vaccine, Zostavax® (shingles (herpes zoster) vaccine (live)) was originally approved by the US Food and Drug Administration and European Medicines Agency for use in patients aged 60 years and above and more recently has gained approval for use in patients aged 50 years or above. That no link was observed between a patient’s age and HZ pain severity and associated impact in the ZQOL highlights the potential value of vaccination in some patients < 60, particularly as it is among these patients where productivity losses may be most apparent.

From the perspective of frontline service provision, findings from the ZQOL study indicate the need for better observation and monitoring of patients treated in primary care to ensure adequate management of HZ. The disconnect evident between patient and doctor assessments of disease severity, especially in terms of severity of pain experienced, indicates the need for doctors to ensure open communication with patients in terms of their current experiences of HZ and PHN. In addition, there appears to be dissatisfaction among HZ patients, especially with regard to the perceived efficacy of treatments, which is presumably driven by experience of inadequate relief of symptoms. Thus, doctor communication of the value of prescribed therapies and setting realistic patient expectations of treatment outcomes may also help to improve adjustment in patients with HZ. Use of standardised PRO questionnaires as utilised in the ZQOL study (e.g. ZBPI and SF-36) may be of value to doctors in clinical practice, both in terms of obtaining symptom and HRQoL reports directly from the patient themselves and also providing patients with a perceived degree of input into their own treatment decisions.

### ZQOL study limitations and opportunities for further research

The UK is a multi-cultural society, with findings from the 2011 UK census indicating that 7.7million (14.0%) of the population in England and Wales belonged to non-white ethnic groups. However, it should be noted that despite recruitment of HZ patients from regional sites throughout the UK, the proportion of non-Caucasian patients in the ZQOL study (0.4%) is lower than UK population estimates and less than reported in prior studies in this area [6,8,10]. That non-English speaking HZ patients were excluded from participation in the study may have contributed to the small number of non-Caucasian patients, but this may also be reflective of observations that HZ is less common among non-Caucasian populations [2]. Nonetheless, as a multi-cultural society, follow-up work may be conducted to explore the burden of HZ as it relates to ethnic minority patients in the UK.

Whilst cross-sectional data generated from the ZQOL study and other studies provides insight into the collective burden experienced by patients with HZ, the lack of long-term follow-up and repeated data collection over time mean that there is limited understanding of the impact of long-term complications of HZ. In particular, research to help further understand factors that may explain the development of PHN in only a subset of HZ patients (and identify those most at risk of developing PHN) and to also understand the impact, at an individual level, that the progression of HZ to PHN has on patients (particularly in terms of pain and HRQoL impact) would be of value.

Research evaluating the burden of the HZ and PHN has, to date, focussed almost exclusively on patient-centred outcomes and costs incurred by healthcare systems and society. However, as a disease most prevalent among those aged 50 years or over, many of whom (based on ZQOL study data) are expected to be living with someone else, it is likely that a significant proportion of patients will receive regular assistance from informal caregivers. The impact on these caregivers providing care to a patient both during the acute presentation and HZ and during any long-term complications (e.g. PHN), however, remains largely unexplored.

Finally, while information collected using formal PRO questionnaires provides a valid means of ‘quantifying’ the burden of HZ, qualitative research exploring the ‘lived experience’ of HZ from a patient’s perspective would be beneficial for further understanding the burden and unmet needs among HZ patients. To the authors’ knowledge, however, no qualitative accounts of the experiences of HZ and PHN within the scientific literature have been published to date.

## Conclusions

As the largest and first UK-wide cross-sectional study conducted in HZ patients, the ZQOL study provides much needed information regarding the characterisation and management of HZ and the resulting burden associated with the condition from a patient and wider societal perspective. The data from this study offer further support to the characterisation of HZ as a painful condition that can have a significant impact on patients’ lives and result in significant costs for healthcare providers. Data from this study also highlight the current unmet need and inadequacy of current treatments in HZ patients, reinforcing the need for effective preventative strategies (such as vaccination) and early intervention.

## Abbreviations

EQ-5D: EuroQol-5 dimensions; HSI: Health state index; HRQoL: Health-related quality of life; HZ: Herpes zoster; HZO: Herpes zoster ophthalmicus; MSC: Mental component summary; NHS: National health service; OLS: Ordinary least squares; PCS: Physical component summary; PHN: Post-herpetic neuralgia; PRO: Patient-reported outcome; QIPP: Quality, innovation, productivity and prevention; SAP: Statistical Analysis Plan; SF-36: Study short-form 36; SRH: Self-rated health status; TSQM: Treatment satisfaction with medication; VZV: Varicella-zoster virus; ZBPI: Zoster brief pain inventory; ZQOL: Zoster quality of life.

## Competing interests

AG and LA are employees of Adelphi Values, a health outcomes agency commissioned by Sanofi Pasteur MSD, to conduct, analyse and communicate findings from this research on their behalf. SC and AM are employees of Sanofi Pasteur MSD, a provider of a herpes zoster vaccine approved in the European Union. RJ has received consultancy and lecture fees from Sanofi Pasteur MSD, Merck and Merck Frosst. MS has no competing interests to declare.

## Author contributions

AG and LA led the design and conduct of the study, analysis and interpretation of study findings and drafting of the manuscript. SC facilitated the conduct of the study, analysis and interpretation of study findings and drafting of the manuscript. AM, RJ & MS contributed to study design, interpretation of study findings and review of the manuscript from a clinical perspective. All authors read and approved the final manuscript.

## Pre-publication history

The pre-publication history for this paper can be accessed here:

http://www.biomedcentral.com/1471-2334/14/402/prepub
